# Microfluidic Device for Screening for Target Cell-Specific Binding Molecules by Using Adherent Cells

**DOI:** 10.3390/mi10010041

**Published:** 2019-01-09

**Authors:** Maho Kaminaga, Tadashi Ishida, Tetsuya Kadonosono, Shinae Kizaka-Kondoh, Toru Omata

**Affiliations:** 1 Department of Mechanical Engineering, School of Engineering, Tokyo Institute of Technology, Kanagawa 226-8503, Japan; ishida.t.ai@m.titech.ac.jp (T.I.); omata.t.aa@m.titech.ac.jp (T.O.); 2 Department of Life Science and Technology, School of Life Science and Technology, Tokyo Institute of Technology, Kanagawa 226-8503, Japan; tetsuyak@bio.titech.ac.jp (T.K.); skondoh@bio.titech.ac.jp (S.K.-K.)

**Keywords:** microfluidic device, target cell-specific binding molecules, screening, adherent cells, pneumatic microvalve, cell homogenous dispersion structure

## Abstract

This paper proposes a microfluidic device for screening molecules such as aptamers, antibodies, proteins, etc. for target cell-specific binding molecules. The discovery of cancer cell-specific binding molecules was the goal of this study. Its functions include filtering non-target cell-binding molecules, trapping molecules on the surface of target cells, washing away unbound molecules, and collecting target cell-specific binding molecules from target cells. These functions were effectively implemented by using our previously developed micro pillar arrays for cell homogeneous dispersion and pneumatic microvalves for tall microchannels. The device was also equipped with serially connected filter chambers in which non-target cells were cultured to reduce the molecules binding to non-target cells as much as possible. We evaluated the performance of the device using cancer cell lines (N87 cells as target cells and HeLa cells as non-target cells) and two fluorescent dye-labeled antibodies: Anti-human epidermal growth factor receptor 2 (anti-HER2) antibody that binds to target cells and anti-integrin antibody that binds to non-target cells. The results showed that the device could reduce anti-integrin antibodies to the detection limit of fluorescent measurement and collect anti-HER2 antibodies from the target cells.

## 1. Introduction

Anti-cancer drugs are widely used for cancer treatment. Conventional anti-cancer drugs not only damage cancer cells but also harm normal cells [1]. One approach for suppressing the damage caused to normal cells is to deliver combinations of cancer cell-specific binding molecules and anti-cancer drugs that act only on cancer cells [2]. Some cancer cell-specific binding molecules have been identified. For example, humanized anti-HER2 antibody (trastuzumab) [3] is used in clinical applications. Cancer cell-specific binding molecules such as cyclic arginine-glycine-aspartic acid tripeptide that specifically binds to malignant brain tumor cells in glioma [4], an aptamer that specifically binds to ovarian cancer cells [5], and a protein that binds to the protein disulfide isomerase, which is highly expressed on the surface of tumor cells [6] have been reported. However, few combinations of cancer cell-specific binding molecules and targets are known. The search for combinations is necessary and is performed by the screening of molecular libraries. To screen for cancer cell-specific binding molecules using cancer cells, conventional screening procedures involve the following steps: (Step 1) normal cells and the molecular library are mixed to filter out molecules that bind to normal cells; (Step 2) unbound molecules and target cancer cells are mixed to capture target cancer cell-specific binding molecules; (Step 3) washing of the target cancer cells; (Step 4) collecting bound molecules; and (Step 5) amplifying collected molecules. These amplified molecules are used in the next round of screening. Cancer cell-specific binding molecules are condensed by repeating steps 1 to 5. In addition to these complicated steps, this screening procedure requires precise manual operations, which are laborious and time-consuming. To conduct screening without human errors and decrease the screening time, automation with precise manipulation is required.

Microfluidic technology is suitable for automation owing to the following advantages: (1) Easy manipulation of liquid and cells owing to the dimensions as small as the size of the cells, (2) multiple processing capability on a single chip, (3) low sample and reagent consumption. Because of these advantages, several microfluidic screening devices have recently been developed. The microfluidic phage selection (MiPS) device can perform the screening using adherent cells [7]. Because this device does not filter the peptides that bind to normal cells, it collects not only cancer cell-specific binding peptides but also non-specific binding peptides. The cell-systematic evolution of ligands by exponential enrichment (Cell-SELEX) chip can isolate target cell-binding single strand deoxyribonucleic acids (ssDNAs) from a combinatorial ssDNA library [8]. The device uses magnetic microbeads attached to the cell surface and traps the beads, and consequently the attached cells, with a magnet. The chambers of normal cells and target cells are serially connected to search for target cell-specific binding ssDNA using a small quantity of ssDNA binding to normal cells in a single chip. Screening devices [9,10,11] also attach magnetic beads on cells to manipulate them under floating conditions. However, the molecules present on the surface of such cells may differ from those present under adherent conditions. Microfluidic devices developed in previous studies do not satisfy the following points: (1) Adherent culture conditions for adherent cells rather than floating condition and (2) filtering out the molecules that bind to non-target cells, which cause side effects.

Therefore, the purpose of this paper was to develop a microfluidic device for removing non-target cell-binding molecules to select target cell-specific binding molecules by using adherent cells in an adhered state. Our microfluidic device can perform steps 1 to 4 of the screening procedure in one chip. Another possible application of the microfluidic device is to detect changes in the expression of molecules depending on the malignancy of cancer cells. For example, the microfluidic device can introduce normal cancer cells as non-target cells and cancer stem cells as target cells. Additionally, if the microfluidic device introduces primary cells obtained from patients, cancer cell-specific binding molecules can be searched for every patient, leading to custom treatment.

For effective filtering, the cultured cells should be uniformly distributed. The cell chambers integrate our previously developed micro pillar arrays (MPAs) [12]. The MPA generates random flow caused by a repetitive cell clog-and-release process at the gaps between the micro pillars, resulting in a uniform distribution of cells. To perform steps 1 to 4 in the microfluidic device, microvalves that open and close the microchannels are necessary. The height of the microchannels is 50 μm, which is relatively tall and enables cells to pass through while minimizing physical interactions in the microchannels. A microvalve fabricated by a reflow process can open and close a microchannel of 50 μm in height. However, a reflow process is not compatible with the fabrication of the MPA, as it makes the shape of the micro pillars round. Therefore, the microfluidic device implements our previously developed pneumatic valves fabricated by using inclined lithography [13]. We evaluated the performances of the developed microfluidic device using known combinations of cells and antibodies, which was necessary to achieve the goal of this study of screening by using molecular libraries to discover cancer cell-specific binding molecules.

This paper is organized as follows: Section 2 describes the fabrication process, design, and operation of the microfluidic device, Section 3 describes evaluation of the performance of the filtering anti-integrin antibody that binds to non-target cells and the performance of collecting HER2 antibodies bound to target cells. Section 4 concludes this paper.

## 2. Materials and Methods 

### 2.1. Microfluidic Device for Screening for Target Cell-Specific Binding Molecules

#### 2.1.1. Fabrication Process of the Microfluidic Device

**Figure 1:**
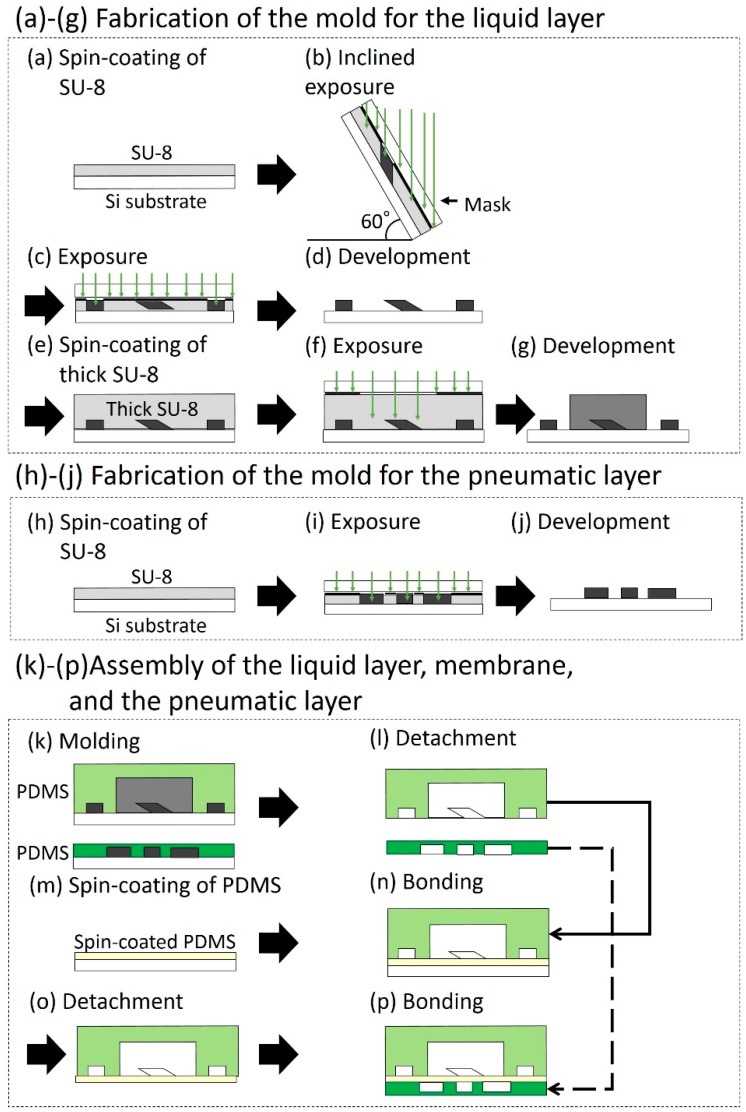
Fabrication process of the microfluidic device. (**a**–**g**) Fabrication of the mold for the liquid layer. (**h**–**j**) Fabrication of the mold for the pneumatic layer. (**k**–**p**) Assembly of the liquid layer, membrane, and the pneumatic layer.

The microfluidic device consisted of three layers: A layer for liquid channels (liquid layer for short), a thin membrane, and a layer for pneumatic channels (pneumatic layer for short). The pneumatic and liquid channels were crossed at the position of the microvalves. All the layers of the microfluidic device were fabricated by soft lithography, and the fabricated layers were bonded to each other (Figure 1). The microchannels in the liquid layer had a parallelogram-shaped cross section, which could be obtained by inclined photolithography.

**Figure 2:**
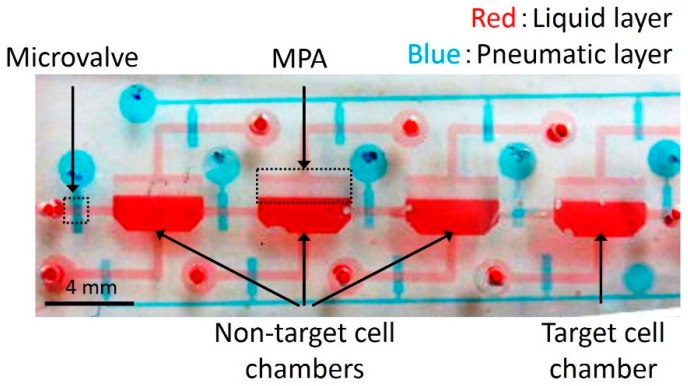
Photograph of the fabricated microfluidic device. The liquid layer was filled with red dye and the pneumatic layer blue dye.

The complete fabrication process is as follows: (a) SU-8 (SU-8 3025, Microchem, Westborough, MA, USA) was spin-coated on a Si substrate. (b) A photomask of the liquid channels was aligned on the Si substrate. The substrate with the photomask was inclined at 60 degrees and exposed to ultra violet (UV) light [13]. (c) A photomask of the MPAs was aligned on the substrate and exposed to UV light without inclining the substrate [12]. (d) The unexposed SU-8 was etched away by the development process, resulting in the mold of the liquid microchannel. (e) Thick SU-8 (SU-8 2150, Microchem) was spin-coated on the developed substrate. (f) A photomask of the microchambers was aligned and exposed to UV light without inclining the substrate. (g) After the development, the patterned thick SU-8 was obtained. (h–j) The mold for the pneumatic microchannels was fabricated by photolithography without inclining the substrate. (k) polydimethylsiloxane (PDMS; Silpot 184 W/C, Dow Corning Toray, Tokyo, Japan. Base polymer to curing agent ratio was 10:1 by weight.) was casted on the molds of the liquid and pneumatic layers. (l) The PDMS structures for the liquid and pneumatic layers were detached from their molds. (m) A thin membrane between liquid and pneumatic layers was fabricated by spin-coating PDMS on a flat Si substrate. (n) The PDMS structure for the liquid layer was bonded to the thin membrane on the flat Si substrate by the surface activation of vacuum UV irradiation. (o) The bonded PDMS structure was detached from the Si substrate. (p) The bonded PDMS structure was again bonded to the pneumatic layer. Figure 2 shows the fabricated microfluidic device. The liquid and pneumatic microchannels were filled with red and blue dyed water, respectively, to improve visibility. 

#### 2.1.2. Design of the Microfluidic Device

**Figure 3:**
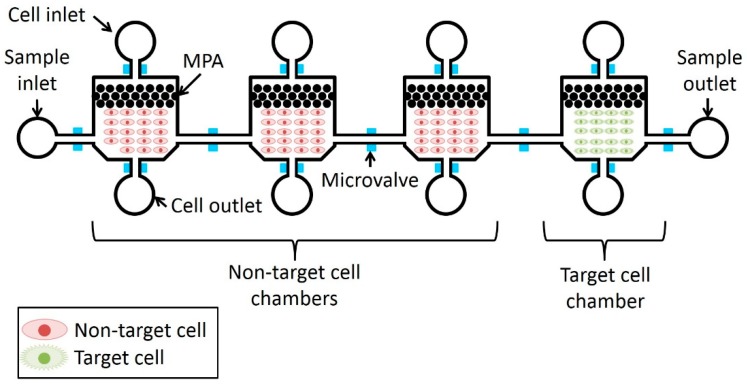
Schematic illustration of the microfluidic device.

A schematic illustration of the microfluidic device is shown in Figure 3. It has chambers capable of adhesively culturing both non-target and target cells. The three chambers upstream that contain non-target cells capture non-target cell-binding molecules and then reduce contamination of non-target cell-binding molecules into the target-cell chamber. The reason for introducing three non-target cell chambers is described in Section 2.1.3. 

For effective filtering, the cultured cells should be uniformly distributed. For this purpose, MPAs are equipped between the cell inlet and the cell chamber [12]. The chambers are gently connected by the microchannels between them to prevent molecules from remaining at the corners of the chamber. Pneumatic microvalves developed in [13] were used to open and close all the microchannels. Each pneumatic microvalve can be independently driven by applying compressed air separately. 

**Figure 4:**
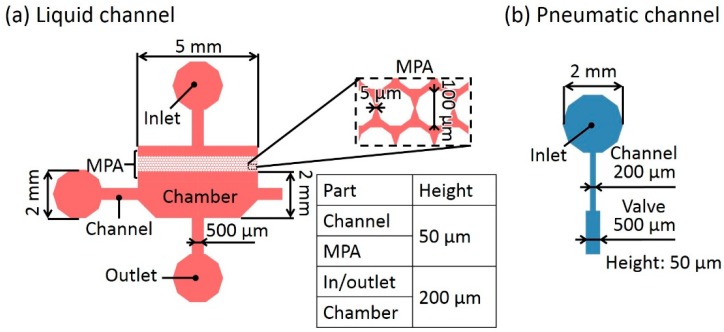
Dimensions of the microfluidic device. (**a**) Liquid channel. (**b**) Pneumatic channel.

Figure 4a shows the dimensions of the liquid channel in the microfluidic device. The length and width of the chamber excluding MPA were 5 mm and 2 mm, respectively. The height of the chambers was set to 200 μm to facilitate cell culture. To prevent the introduction of bubbles entering the liquid channels, the heights of the inlet and outlet were also set to 200 μm, whereas the liquid channels connected to the chamber was 500 μm in width and 50 μm in height for easy passage of the cells and closing of the microvalves [13]. The diameter and interval of the micro pillars were 100 μm and 5 μm, respectively. The thickness of the thin membrane was 40 μm. Figure 4b shows the dimensions of the pneumatic channel to control the microvalves. 

#### 2.1.3. Operation of the Microfluidic Device

**Figure 5:**
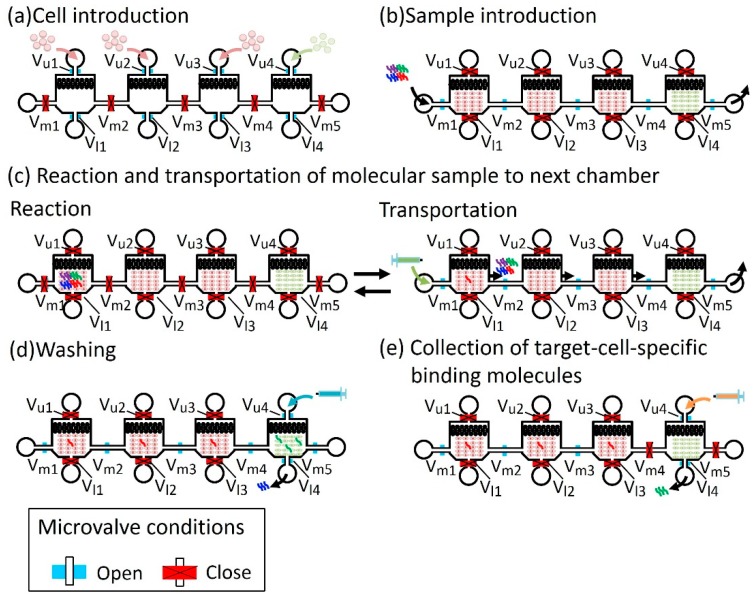
Operation of the microfluidic device to perform the screening procedure from step 1 to step 4. (**a**) Cell introduction. (**b**) Sample introduction. (**c**) Reaction and transportation of molecular sample to the next chamber. (**d**) Washing. (**e**) Collection of target-cell-specific binding molecules.

Figure 5 shows the operation of the microfluidic device that performed the screening procedure from steps 1 to 4. Open and closed microvalves are shown in blue and red, respectively. (a) Cell Introduction: Non-target and target cells were introduced into the chambers from the cell inlets, (Figure 5a). In this step, microvalves at the cell inlets—V_u1_, V_u2_, V_u3_, and V_u4_—and microvalves at the cell outlets—V_l1_, V_12_, V_l3_, andV_l4_—were open, and the microvalves between the cell chambers—V_m1_, V_m2_, V_m3_, V_m4_, and V_m5_—were closed to prevent cross-contamination of cells. The chambers cultured the introduced cells, maintaining the microvalve condition. (b) Sample introduction: A molecular sample (or library in the case of screening) was introduced from the molecular sample inlet into the non-target cell chamber (Figure 5b). The microvalve condition becomes opposite; microvalves V_u1_, V_u2_, V_u3_, V_u4_, V_l1_, V_12_, V_l3_, and V_l4_ were closed to prevent the leakage of the molecular sample, and microvalves V_m1_, V_m2_, V_m3_, V_m4_, and V_m5_ were kept open. (c) Reaction and transportation of the molecular sample to the next chamber: Non-target cell-binding molecules were captured in each of the non-target cell chambers in a certain reaction time. During the reaction, microvalves V_m1_, V_m2_, V_m3_, V_m4_, and V_m5_ were closed. Introducing oil from the sample inlet transfers the sample to the next chamber. During the transportation, microvalves V_m1_, V_m2_, V_m3_, V_m4_, and V_m5_ were open and other microvalves were closed (Figure 5c). This step was repeated until the molecular sample arrived at the target cancer cell chamber. (d) Washing: The unbound molecules were washed by introducing a washing buffer from the cell inlet of the target cell chamber (Figure 5d). In this step, microvalves V_u4_, V_l4_, V_m1_, V_m2_, V_m3_, V_m4_, and V_m5_ were open and the others were closed. (e) Collection of target cell-specific binding molecules: The specific molecules that bound to target cells were collected by introducing a collection buffer (Figure 5e). Only microvalves V_u4_ and V_l4_ were open and the others were closed to prevent contamination of molecules from one chamber to another.

Note: While introducing a sample solution, it pushes out the culture medium filling the chamber. If the chamber volume is larger than the sample volume, the remaining culture medium in the chamber dilutes the sample solution. Therefore, the chamber volume must be the same as the sample volume. The number of non-target cell chambers (not its volume) is the factor that can be altered to improve the filtering performance. In this study, the number of non-target cell chambers was three. This was because a microfluidic device with only one non-target cell chamber did not achieve adequate performance of filtering in our preliminary experiments. Furthermore, when the number of the chambers exceeded four, the reaction time also exceeded by 10 h, which was too long for one cycle of screening.

#### 2.1.4. Cells

The human gastric carcinoma cell line N87 and human cervical cancer cell line HeLa were purchased from the American Type Culture Collection (Manassas, VA, USA). N87 cells were used as target cells and HeLa cells were used as non-target cells (Table 1). 

N87 cells express HER2 (target molecules) and HeLa cells do not, while both the cell lines express integrin. Both the cell lines were cultured in Dulbecco’s Modified Eagle’s Medium (DMEM; D-MEM (High Glucose) with L-Glutamine, Phenol Red and Sodium Pyruvate, Wako, Osaka, Japan) supplemented with 10% fetal bovine serum (FBS; Fetal Bovine Serum regular, Wako) and 100 UI/mL penicillin–streptomycin (Penicillin-Streptomycin solution (×100), Wako). They were incubated at 37 °C under 5% CO_2_ condition. Cell suspensions were made by detaching cells from the cell culture dish, and mixing with DMEM. Both N87 and HeLa cells were detached using trypsin solution (0.25 w/v% Trypsin Solution with Phenol Red, wako) at room temperature for 10 min or 2 min, respectively.

**Figure 6:**
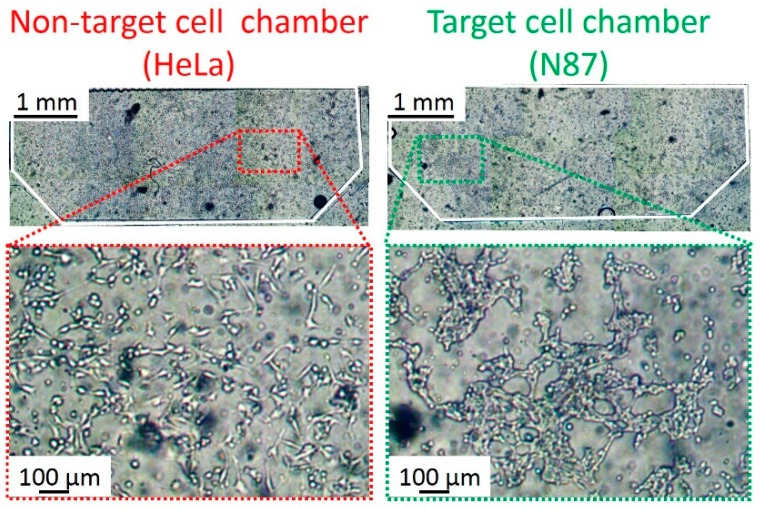
Micrographs of the HeLa and N87 cells cultured in the cell chambers. The HeLa and N87 cells were separately cultured in non-target and target cells without cross contamination.

Figure 6 shows the HeLa and N87 cells cultured inside the chambers. The N87 cell suspension (1 × 10^4^ cells/μL) was introduced into the target cell chamber at 2 μL/min for 1 min, and incubated for 6 h at 37 °C under 5% CO_2_ condition. The HeLa cell suspension (1 × 10^4^ cells/μL) was introduced into the non-target cell chambers at 2 μL/min for 1 min, and incubated for 6 h at 37 °C under 5% CO_2_ conditions. Owing to the MPA, both HeLa and N87 cells spread to the full width of the chambers. Besides, cell morphology in the chambers indicated that there was no contamination of the cells due to the pneumatic microvalve between the chambers. Video S1 shows that cells did not flow into the adjacent chamber by closing the valve. After washing the chambers with phosphate buffer salts (PBS, Wako) at 10 μL/min for 10 min, the cells were fixed by using the 4% paraformaldehyde solution (4%-Paraformaldehyde Phosphate Buffer Solution, Nacalai Tesque, Kyoto, Japan) (20 °C for 15 min). In this study, fixation was necessary because the antigen-antibody binding took a long time, which made the cells in the chambers inactive or mortal. After the fixation, blocking was performed using 5% fetal bovine serum -PBS (20 °C for 60 min). 

#### 2.1.5. Reagent

Fluorescent dye-labeled antibodies were used as binding molecules. Anti-HER2 antibody conjugated to AF 488(Alexa Fluor^®^ 488 anti-human CD340 (erbB2/HER2) Antibodies, BioLegend, San Diego, CA, USA) was used as an antibody that specifically binds to target cells (abbreviated as target-specific Ab). AF 488 dye has 490 nm in excitation wavelength and 525 nm in emission wavelength (Green fluorescence). Anti-integrin antibodies conjugated to AF 555 (Anti-integrin α_v_β_5_ Antibody, Alexa Fluor^®^ 555 Conjugated, Bioss, Woburn, MA, USA) was used as an antibody that binds to non-target cells (abbreviated as non-specific Ab). AF 555 dye has 555 nm in excitation wavelength and 580 nm in fluorescent wavelength (Red fluorescence) (Table 2). The concentration of the non-specific antibody (Ab) solution was 10 μg/mL, and that of target-specific Ab was 4 μg/mL. The mixture showed similar fluorescence intensity. The reaction time for both Abs was 2 h at 37 °C. Canola oil (cooking canola oil, Ajinomoto, Tokyo, Japan) was used for sample transportation. It contained 64.0% of oleic acid, 20.6% of linoleic acid, 9.5% of alpha-linolenic acid, 4.1% of palmitic acid, and 1.8% of stearic acid. The mixture of 1% Sodium Dodecyl Sulfate (SDS; Sodium Dodecyl Sulfate, Wako), 0.1% Tween 20 (Polyoxyethylene Sorbitan Monolaurate, TCI, Tokyo, Japan), and PBS was used as a collection buffer, which was similar to the collection buffer described in the T7 phage screening protocol [14].

### 2.2. Experimental Procedure

#### 2.2.1. Experimental Setup

The experiments in this paper were conducted under a fluorescence microscope (IX-83, Olympus, Tokyo, Japan). The fluorescence intensities of the cells in the chambers were measured using a fluorescence microscope. Filters U-FGWA (Olympus) and U-FBNA (Olympus) were used for red and green fluorescence imaging, respectively. The fluorescence intensity of a solution was measured using a flourometer (Infinite F500 microplate reader, Tecan, Männedorf, Switzerland) with Ex/Em filters (485 ± 20 nm/ 535 ± 25 nm for AF488 or 535 ± 25 nm/ 590 ± 20 nm for AF555). A syringe pump (KDS-210, KD scientific, Holliston, MA, USA) was connected to the inlets of the microfluidic device to introduce cells, fluorescent dye-labeled antibodies, culture medium, and PBS. A pneumatic pressure source (OFP-07005, Iwata, Kanagawa, Japan) was connected to the inlets of pneumatic channels of the microfluidic device via a solenoid valve array (SY114-5LZ, SMC, Tokyo, Japan) and a regulator (IR1020-01BG-A, SMC) to switch the microvalves. 

#### 2.2.2. Filtering Non-Specific antibodies (Abs)

**Figure 7:**
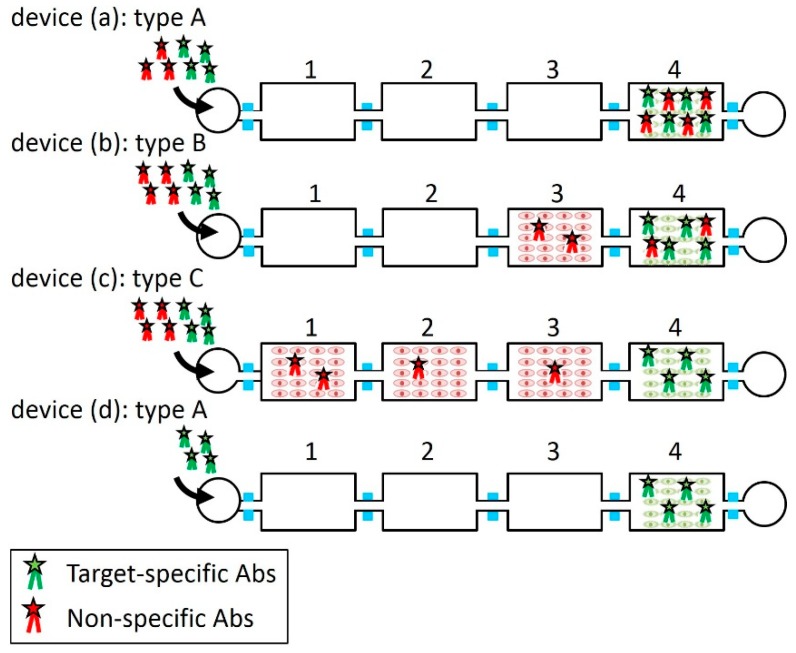
Four microfluidic devices with three types for the experiment of filtering the non-specific antibodies (Abs). The mixture of the fluorescent dye-labeled target-specific Ab and non-specific Ab solutions was introduced to the devices. (**a**) Type A: Three blank and one target cell chambers. (**b**) Type B: Two blank, one non-target cell and one target cell chambers. (**c**) Type C: Three non-target and one target cell chambers. (**d**) Type A: Only target-specific Ab solution was introduced for autofluorescence measurement.

The performance of the filtering, which removes non-specific Abs, was examined. We prepared four microfluidic devices (devices (a), (b), (c) and (d)) of three different types as shown in Figure 7: (type A) Three blank chambers and one target cell chamber; (type B) two blank chambers, one non-target cell chamber and one target cell chamber; (type C) three non-target cell chambers and one target cell chamber. The chambers of each microfluidic device were numbered 1, 2, 3, and 4 on the upstream side. 

The performance of the filtering can be assessed by the amount of non-specific Abs bound to the target cancer cells. The mixture of the fluorescent dye-labeled target-specific Ab and non-specific Ab solutions were introduced to devices (a), (b) and (c) at 2 μL/min for 1 min in the same operations. As the number of non-target cell chambers increased, it was expected that they filtered more non-specific Abs and red fluorescence intensity decreased in the target cell chamber. The ratio of red to green fluorescence intensities per unit area from target cells was used to evaluate the performance of the filtering. For the autofluorescence measurement, only target-specific Ab solution was introduced to type A device (d) at 2 μL/min for 1 min. The temperature of the chambers was maintained at 37 °C for 2 h. Introducing canola oil from the inlet at 2 μL/min for 1 min transported the solution to the next chamber. This operation was repeated until the mixture or solution reached chamber 4. The mixture or solution was kept in chamber 4 for 2 h. Then, chamber 4 was washed off using PBS at 100 μL/min for 10 min. After washing chamber 4, the fluorescence image of chamber 4 was observed using the fluorescence microscope.

#### 2.2.3. Collecting Target-Specific Antibodies (Abs)

The target-specific Abs on the surface of the target cells need to be collected for amplification or identification for screening. To detach the target-specific Abs from the target cells, a collection buffer was introduced into the target cell chamber. To evaluate the collection method, the fluorescence intensities of the target cells and the collected solution were measured. The detachment of the target-specific Abs on the target cells was measured by comparing the fluorescence intensity before the detachment with that after the detachment. It was expected that the fluorescence intensity of the target cells decreased and that of the collected solution increased.

We prepared four microfluidic devices for this experiment and used only the target cell chamber of each device (chamber 4). Three of them were used to find the sufficient reaction time and one was used as a control. A target-specific Ab solution was introduced into the target cell chamber (4 μg/mL) at 2 μL/min for 1 min and incubated for 2 h at 37 °C. Subsequently, unbound antibodies were washed by PBS at 100 μL/min for 10 min. The target cell chamber was filled with the collection buffer (PBS for the control device) at 2 μL/min for 1 min. The reaction times were 15 min, 30 min, and 60 min, respectively, at 20 °C to find the sufficient reaction time. After the reaction, the buffer in the target cell chamber was collected from the cell outlet by PBS at 100 μL/min for 5 min. 

## 3. Results and Discussion

### 3.1. Results of Filtering Non-Specific Antibodies (Abs)

**Figure 8:**
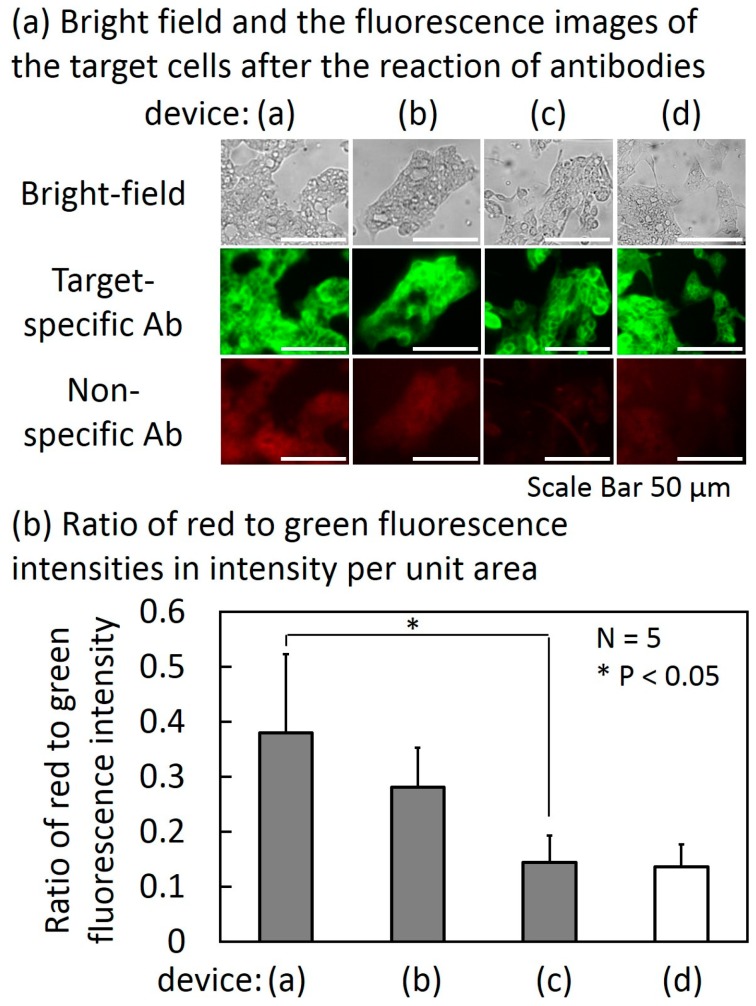
Bright field and the fluorescence images of the target cells (N87 cells) after the reaction of antibodies (**a**) and the ratio of red to green fluorescence intensities in intensity per unit area (**b**).

Figure 8 shows the results of the experiment of filtering non-specific Abs described in Section 2.2. Figure 8a shows the bright-field and fluorescence images of the target cells in the target cell chambers of devices (a), (b), (c) and (d) and Figure 8b shows the ratio of red and green fluorescence intensities of the target cells in intensity per unit area. The result of the autofluorescence measured in device (d) was a control.

In device (a), which had no non-target-cell chambers, the average red to green ratio (*N* = 5) was 0.38. The ratio decreased as the number of the non-target cell chambers increased. In the cases of one non-target cell chamber and three non-target cell chambers (devices (b) and (c), respectively), the average red to green ratios were 0.28 and 0.14, respectively. The reduction ratio in the case of three non-target cell chambers reached 63% in comparison with no non-target cell chamber. The red to green ratio was comparable to that of the autofluorescence measurement in device (d). The result suggests that the device was able to reduce the non-specific Abs to the detection limit of the fluorescent measurement (smaller than 2 μg/mL as shown in Figure S1) when the number of the non-target cell chambers was three. However, the non-specific Abs may not have been completely removed because fluorescence weaker than autofluorescence cannot be measured. Because the cells were fixed with 4% paraformaldehyde solution, non-target cells did not float and drift into the next chamber during sample transportation as shown in Video S2.

The MPAs will influence the binding procedure of molecules in the future study of screening. Because the oil pushed the sample solution from the sample inlet, the sample around the micro pillars remained between the cell inlet and cell culture area in the chamber without flowing into the next chamber. Partial loss of the sample molecules did not affect the results of this paper because known antibodies were used in the experiments. However, this sample transportation method should be improved for screening using a molecular library. By introducing oil from the cell inlet, the remaining sample around the micro pillars can be pushed out.

### 3.2. Results of Collecting Target-Specific Antibodies (Abs)

**Figure 9:**
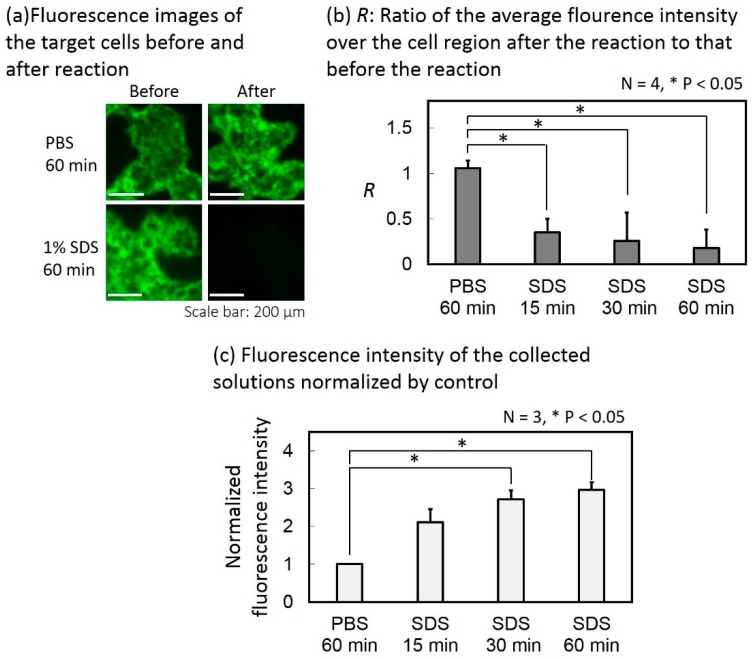
Collection of the target-specific antibodies (Abs) attached on the target cells. (**a**) Fluorescence images of target cells before and after the reaction. (**b**) Ratio of the average fluorescence intensity over the cell region after the reaction to that before the reaction. (**c**) Fluorescence intensities of the collected solutions normalized by control.

The fluorescence images of the target cells before and after the reaction with the collection buffer are shown in Figure 9a. The reaction time was 60 min. The average fluorescence intensity over the cell region was calculated for each fluorescence image and the ratio of that after the reaction to that before the reaction (denoted as *R*) was calculated. Figure 9b shows the results. When only PBS was introduced instead of the collection buffer, *R* was 1.06. When the collection buffer filled the target cell chamber for 15 min, 30 min and 60 min for reaction, *R* was 0.35, 0.26, and 0.18, respectively. According to a student’s test, *R* significantly decreased in all reaction times between the target cells and the collection buffer, compared with the case where only PBS was introduced.

The fluorescence intensity of the solution collected from each device was measured and normalized by that of the control device. Figure 9c shows the results. When the collection buffer filled the target cell chamber for 15 min, 30 min and 60 min for reaction, respectively, the average fluorescence intensities of the collected solutions (*N* = 3) were 2.1, 2.7, and 3.0 times higher than the case where only PBS was used instead of the collection buffer. According to a Student’s T-test, the fluorescence intensity increased significantly when the reaction times were 30 min and 60 min compared with the case where only PBS was introduced. 

From Figure 9b,c, the fluorescence intensity measured from the collected solution increased when the fluorescence intensity of the target cells decreased, as the reaction time increased. Therefore, the target-specific Abs bound on the surface of the target cells were successfully detached and collected.

### 3.3. Future Prospects on the Development of the Device

The microfluidic device proposed here carries out molecular selection using two-dimensionally cultured cells. However, differences between in vitro and in vivo environments are a general problem in the screening of target cell-specific binding molecules. For clinical applications, the screening results must be very precise. Investigation of cell culture methods is necessary for improving precision.

In recent years, many techniques for three-dimensional cell culture have been developed. It is said that such cell models are closer to the in vivo environment. More recently, a mesh cell culture method was proposed [15], which used a mesh for a scaffold. According to [15], it can reduce cell-substrate adhesion and promote cell-cell adhesion, thus the cell model is closer to the in vivo environment. This device can easily incorporate the mesh cell culture method by modifying the substrates of the chambers.

## 4. Conclusions

We developed a microfluidic device to screen for molecules that specifically bind to target cells by filtering nonspecifically binding molecules. By using the pneumatic microvalves, the microfluidic device was able to perform the following functions: Filtering of non-target cell-binding molecules, trapping of molecules on the surface of target cells, washing of unbinding molecules, and collecting target cell-specific binding molecules from the target cells. We evaluated the performance of the microfluidic device using cancer cell lines and fluorescent dye-labeled antibodies. The results showed that the microfluidic device could reduce the antibodies that bound to non-target cells to the detection limit of fluorescent measurement and could collect antibodies that specifically bound to target cells. To perform the screening in the microfluidic device, conditions such as the blocking conditions, reaction time, and collection buffer must be optimized. In future studies, we will conduct screening experiments using molecular libraries to identify effective cancer cell-specific binding molecules. 

## Figures and Tables

**Table 1:** Cell lines used in the experiments.

	Name of Cell Lines	Expressing Surface Molecules	
**Target cells**	N87 cell lines	HER2 (Target molecules)	Integrin α_v_β_5_
**Non-target cells**	HeLa cell lines	-	Integrin α_v_β_5_

**Table 2:** Antibodies used in the experiments.

	Name of Molecules	Binding Cells		Fluorescent Dye
**Target cell-specific binding molecules**	Anti-HER2 antibody (Target-specific Ab)	N87 cells (Target cells)	-	AF 488 (Green fluorescence)
**Non-target cell-binding molecules**	Anti-inegrin antibody (Non-specific Ab)	N87 cells (Target cells)	HeLa cells (Non-target cells)	AF 555 (Red fluorescence)

## Supplementary Materials

The following are available online at http://www.mdpi.com/2072-666X/10/1/41/s1, Figure S1: Relationship between concentration of non-specific antibodies and mean fluorescence intensity of the cell area normalized by autofluorescence of the target cells. Video S1: Non-target cell chamber in the cell introduction. Cell suspension was introduced into the chamber from the cell inlet. Because the valves between the chambers were closed, the cells introduced from the cell inlet did not flow into the next chamber, but rather into the cell outlet. Video S2: Non-target cell chambers during sample transportation. Because the cells were fixed with 4% paraformaldehyde solution, non-target cells did not float and drift into the next chamber. The video is at 4 times speed.
