# Supplementary material for: Microfluidic Device for Screening for Target Cell-Specific Binding Molecules by Using Adherent Cells
Source: Micromachines (Basel). 2019 Jan 9;10(1):41. doi: 10.3390/mi10010041 (PMC6356270; doi:10.3390/mi10010041)
Supplement: Supplementary file 1 [file micromachines-10-00041-s001.zip › micromachines-416746-SI/micromachines-416746-supplementary - english done.docx]

**Supplementary Materials**


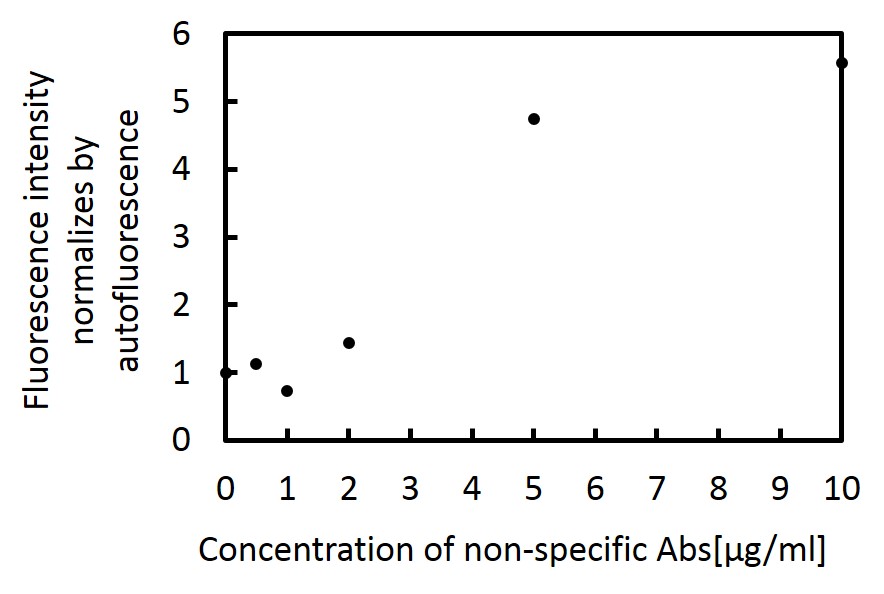


**Figure S1.** Relationship between concentration of non-specific antibodies (Abs) and mean fluorescence intensity of the cell area normalized by autofluorescence of the target cells.

**Video S1.** Non-target cell chamber in the cell introduction. Cell suspension was introduced into the chamber from the cell inlet. Because the valves between the chambers were closed, the cells introduced from the cell inlet did not flow into the next chamber, but rather into the cell outlet.

**Video S2.** Non-target cell chambers during sample transportation. Because the cells were fixed with 4% paraformaldehyde solution, non-target cells did not float and drift into the next chamber. The video is at 4 times speed.
